# Supplementary material for: Outcomes in Pregnant Persons Immunized with a Cell-Based Quadrivalent Inactivated Influenza Vaccine: A Prospective Observational Cohort Study
Source: Vaccines (Basel). 2022 Sep 23;10(10):1600. doi: 10.3390/vaccines10101600 (PMC9612226; doi:10.3390/vaccines10101600)

SUPPORTING INFORMATION FOR

Outcomes in Pregnant Persons Immunized with Cell-Based Quadrivalent Inactivated Influenza Vaccine: A Prospective Observational Cohort Study

Christopher Robinson,[a,*] Josephine Van Boxmeer,[b,*] Hugh Tilson,[c] Anthony Scialli,[d] John A. Vanchiere,[e] Ellis Ides,[b] Daphne Sawlwin,[f] Deborah Molrine,[g] Matthew Hohenboken,[g] Jonathan Edelman,[h] and Jessica D. Albano[i]

[a] Charleston Maternal Fetal Medicine, Mount Pleasant, South Carolina, USA [b] Seqirus Netherlands B.V., Amsterdam, the Netherlands [c] Gillings School of Global Public Health, University of North Carolina, Chapel Hill, North Carolina, USA [d] Scialli Consulting LLC, Washington DC, USA [e] Louisiana State University Health Science Center, Shreveport, Louisiana, USA [f] Seqirus Pty Ltd, Parkville, Victoria, Australia [g] Seqirus USA Inc., Cambridge, Massachusetts, USA [h] Seqirus USA Inc., Summit, New Jersey, USA [i] Syneos Health, Wilmington, North Carolina, USA

*These authors contributed equally to the work and should be considered as first authors.

Study Population Selection

The minimum eligibility criteria required for enrollment were as follows:

- Sufficient information to confirm that the exposure of interest occurred during pregnancy and at which date.
- Sufficient information to determine whether it concerned a prospectively enrolled person, since retrospective cases were ineligible for enrollment.
- Reporter (eg, HCP) contact information was required to allow for follow-up.

Recruitment Strategies

Study enrollment was open to all eligible pregnant persons; however, a number of activities were implemented to increase the enrollment rate. Both passive and active recruitment strategies were implemented. One recruitment strategy targeted HCPs who were known to immunize pregnant persons specifically with the cell-based quadrivalent inactivated influenza vaccine (IIV4c). Also, a targeted awareness campaign was undertaken. This included the distribution of a comprehensive informational kit designed to support HCPs in soliciting interest among pregnant persons in study participation. All messaging was in line with approved US product labeling of IIV4c.

The kit included:

- A brochure that briefly described the study purpose and procedures
- Enrollment form and sample informed consent form
- Important Safety Information
- Participant consent to contact card, which enabled the Coordinating Center to contact the potentially participating persons and provide additional information about the study

A variety of other approaches to provide information to pregnant persons and their HCPs for possible enrollment in the pregnancy registry was also used:

- Internet:
  - FDA listing of pregnancy registries on www.fda.gov
  - www.clinicaltrials.gov
  - Society for Maternal Fetal Medicine listing of registries
  - Immunizationforwomen.org "Monitoring the Safety of Influenza Vaccination during Pregnancy"
  - REPROTOX®
  - Seqirus US website
- Print:
  - IIV4c package insert
  - IIV4c medication guide
- Education:
  - Active outreach to obstetric HCPs using IIV4c routinely in their practice

Table S1. Demographic and baseline characteristics

Characteristic	Primary analysis population (n=665)	Lost to follow-up population (n=27)
Mean age ± SD, years	28.0 ± 5.3	28.9 ± 5.3
Age group, n (%)		
<20 years	18 (2.7)	1 (3.7)
20-24 years	178 (26.8)	5 (18.5)
25-34 years	389 (58.5)	17 (63.0)
35-39 years	68 (10.2)	3 (11.1)
≥40 years	12 (1.8)	1 (3.7)
Race and ethnicity, n (%)		
White	399 (60.0)	13 (48.1)
Black or African American	194 (29.2)	7 (25.9)
Asian	29 (4.4)	5 (18.5)
American Indian or Alaskan Native	1 (0.2)	0
Native Hawaiian or Other Pacific Islander	2 (0.3)	0
Other	28 (4.2)	1 (3.7)
Unknown	12 (1.8)	1 (3.7)
Hispanic or Latino ethnicity	44 (6.6)	1 (3.7)
Mean body mass index ± SD, kg/m^2	29.6 ± 8.2	27.6 ± 6.8
Number of previous pregnancies, n (%)		
0	195 (29.3)	11 (40.7)
1	186 (28.0)	3 (11.1)
2	135 (20.3)	5 (18.5)
≥3	149 (22.4)	8 (29.6)
Family history of congenital malformations, n (%)		
Offspring	8 (1.2)	0
Maternal history	40 (6.0)	2 (7.4)
Paternal history	40 (6.0)	2 (7.4)
Any family history	78 (11.7)	4 (14.8)
Any concurrent condition, n (%)	527 (79.2%)	16 (59.3)
Any concomitant medication, n (%)	651 (97.9)	25 (92.6)
Substance use, n (%)		
Tobacco	84 (12.6)	4 (14.8)
Alcohol	1 (0.2)	1 (3.7)
Illicit drug	0	0
Gestational age at enrollment, n (%)		
<20 weeks	211 (31.7)	19 (70.4)
≥20 weeks	454 (68.3)	8 (29.6)

**Characteristic**	**Primary analysis population (n=665)**	**Lost to follow-up population (n=27)**
Gestational age at IIV4c exposure (days)		
Mean ± SD	152.7 ± 64.0	85.3 ± 34.0
First trimester, n (%)	178 (26.8)	18 (66.6)
Second trimester, n (%)	277 (41.7)	9 (33.3)
Third trimester, n (%)	210 (31.6)	0

Abbreviations: SD, standard deviation.

Figure S1. IIV4c exposure per gestational week for all enrolled participants.

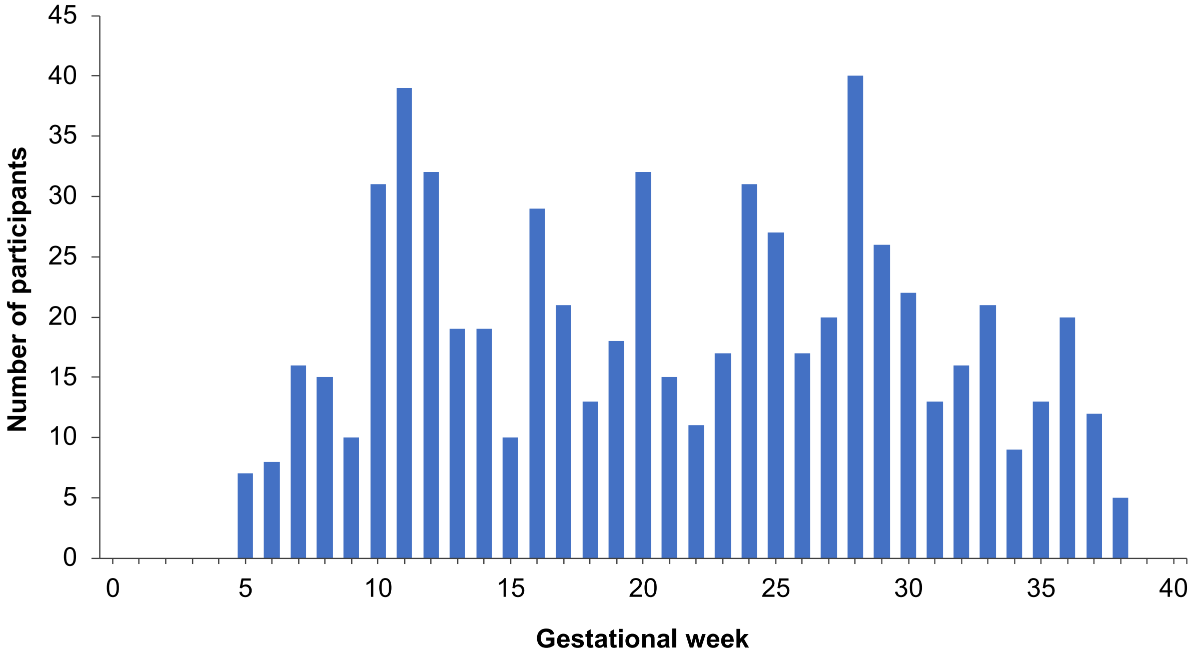

Supplement: Supplementary file 1 [file vaccines-10-01600-s001.zip › vaccines-1877602-supplementary.pdf]
